# When do parts form wholes? Integrated information as the restriction on mereological composition

**DOI:** 10.1093/nc/niad013

**Published:** 2023-06-02

**Authors:** Kelvin J McQueen, Naotsugu Tsuchiya

**Affiliations:** Philosophy Department, Chapman University, California, United States; Turner Institute for Brain and Mental Health & School of Psychological Sciences, Faculty of Medicine, Nursing, and Health Sciences, Monash University, Melbourne, Victoria, Australia; Center for Information and Neural Networks (CiNet), National Institute of Information and Communications Technology (NICT), Suita, Osaka 565-0871, Japan; Computational Neuroscience Laboratories, Advanced Telecommunications Research Institutes International (ATR), 2-2-2 Hikaridai, Seika-cho, Soraku-gun, Kyoto 619-0288, Japan

**Keywords:** mereology, composition question, integrated information theory, IIT, consciousness, feedback connectivity

## Abstract

Under what conditions are material objects, such as particles, parts of a whole object? This is the composition question and is a longstanding open question in philosophy. Existing attempts to specify a non-trivial *restriction* on composition tend to be vague and face serious counterexamples. Consequently, two extreme answers have become mainstream: composition (the forming of a whole by its parts) happens under *no* or *all* conditions. In this paper, we provide a self-contained introduction to the integrated information theory (IIT) of consciousness. We show that IIT specifies a non-trivial restriction on composition: composition happens when integrated information is maximized. We compare the IIT restriction to existing proposals and argue that the IIT restriction has significant advantages, especially in response to the problems of vagueness and counterexamples. An appendix provides an introduction to calculating parts and wholes with a simple system.

## Introduction

The H_2_O molecule is often represented as an oxygen atom and two hydrogen atoms. On a common sense view, the three atoms are *parts* that make up, or compose a further object, the H_2_O molecule. In this example, ‘a further object’ means ‘a fourth object’ since the H_2_O molecule is not identical to any one of the three atoms.

But what about the two hydrogen atoms, do they also compose a further object? Or take a more extreme example. An arbitrary H_2_O molecule on Earth and a quark at the center of the Sun. Do those two objects compose a further object? Common sense would suggest not.

Under what conditions, then, do two or more wholly distinct, or non-overlapping, material objects compose a further, composite object? This is a central question in mereology (the study of part–whole relations) known as *the composition question* [Often it is called the *special composition question* (we remove the qualifier), see van Inwagen (1987, 1990). For an earlier formulation see Hestevold (1981). For an introduction to mereology see Varzi (2019)]. For a question that is relatively easy to state and understand, it has proven terribly difficult to answer.

Democritus (born 450 BC) is said to have announced that there is nothing but atoms in the void (where ‘atom’ translates to ‘indivisible’ or ‘partless’ particle). We will refer to partless particles as ‘simples’. The modern descendant of this view is mereological *nihilism*, which states that composition happens under *no* condition: only simples exist. On this view, the H_2_O molecule does not exist, since if it did exist, it would have parts [For defense of nihilism, see Rosen (2002), Grupp (2006), Liggins (2008), Sider (2013), Contessa (2014)].

Popular among contemporary philosophers is the other extreme, mereological *universalism*, which states that composition happens under *any* conditions: any collection of disjoint objects composes a further object. Thus, not only do the hydrogen and oxygen atoms compose an object (the H_2_O molecule), but so does that molecule and a quark at the center of the Sun. One can consider all kinds of strange objects, such as the object composed of my coffee mug and the King of England. Indeed, full *diachronic* universalism allows all manner of temporally non-local objects, such as the object composed of Queen Elizabeth II and a Tyrannosaurus Rex [For defense of universalism, see Lewis (1986), Lewis (1991), Heller (1990), Jubien (1993), Sider (2001), Hudson (2001), Van Cleve (2008). For an argument that diachronic universalism entails objects moving faster than light, see Hudson (2002)].

The more moderate, common sense answer to the composition question is mereological *restrictionism*, which states that composition happens under only certain non-trivial conditions. Some composites (the H_2_O molecule, perhaps) exist. But it is not the case that any distribution of matter yields a composite. Restrictionism generates a research program: formulate and evaluate hypotheses about the conditions for composition.

Much of the contemporary debate stems from van Inwagen (1990). Van Inwagen’s restriction is surprising: *life*—only living things and simples exist. Van Inwagen argues as follows:

1. Only self-maintaining things are composites.
2. Only living things are self-maintaining.
3. So, only living things are composites.

Both premises (1) and (2) can be challenged. Regarding (2), there has been a long history of failed attempts to define self-maintenance to explain life [For example see the definition in terms of autopoiesis (Varela and Maturana (1980)) and subsequent literature (Razeto-Barry (2012))]. Regarding (1), van Inwagen’s restriction has been criticized on the basis of *counterexamples* (there are no H_2_O molecules, since they do not seem to exhibit the kind of self-maintenance we see in organisms) and *ontological vagueness* (neither ‘self-maintenance’ nor ‘life’ are well-defined) e.g. see (Markosian, 2008, p.351).

As we discuss in the next section, most proposed restrictions on composition face these two objections (counterexamples and ontological vagueness). We therefore aim to propose a restriction that is in a better position to respond to them. Counterexamples to a proposed restriction are cases in which composition occurs according to the proposed restriction but *intuitively* should not occur (or composition does not occur according to the restriction but intuitively should occur). We consider specific cases in detail in section ‘The problem of counterexamples’. Sider (2001, pp.121-32), following Lewis (1986), has developed the problem of ontological vagueness into an influential blanket objection to all non-trivial restrictions. The general concern is that if the restriction is not well-defined, then we will be left with borderline cases of existence. We argue that our proposed restriction goes a long way in solving this problem in section ‘The problem of vagueness’.

Our proposed restriction is related to van Inwagen’s proposal as we shall see. But it is even more closely related to a proposal from Merricks (2001), where the restriction is based on *consciousness*: only conscious things and simples exist. Merricks argues for this as follows:

1. Only causally irreducible things are composites.
2. Only conscious beings are causally irreducible.
3. So, only conscious beings are composites.

According to Merricks, causal irreducibility is obtained when an object has causal powers beyond the powers of its simple parts. However, Merricks does not provide experimental evidence of (2), but instead bases it on an argument for a strong form of mind–body dualism that many have found unpersuasive e.g. Sider (2003).

We agree that self-maintenance and causal irreducibility may be important to understanding how parts form wholes. However, these concepts cannot solve the composition problem until they are more rigorously defined. We propose understanding composition in terms of related concepts, which are formalized by the integrated information theory (IIT) [The IIT is an influential theory of consciousness, founded by Giulio Tononi (see Tononi (2004), Tononi (2008)) and expanded upon by many authors e.g. Oizumi et al. (2014), Tononi (2015), Tononi (2016), Tegmark (2016), Tsuchiya (2017), McQueen (2019a), Mø rch (2019), Haun and Tononi (2019), Barbosa et al. (2020), Ellia et al. (niab032), Grasso et al. (2021), Chalmers and McQueen (2022)].

After formally defining the composition question (section ‘Formal statement of the composition question’), we define causal irreducibility in terms of integrated information (section ‘From causal irreducibility to integrated information’) and we connect self-maintenance to feedback connectivity (section ‘From self-maintenance to feedback connectivity’). We then argue that IIT allows us to calculate and measure composite systems and so makes significant progress on solving the problems of ontological vagueness (section ‘The problem of vagueness’) and serious counterexamples (section ‘The problem of counterexamples’). Finally (section ‘Conclusion’), we discuss philosophical implications for consciousness and existence, and whether they should be understood as coming in degrees. An appendix provides an introduction to calculating parts and wholes with a simple system.

## Formal statement of the composition question

The following formulation of the composition question is inspired by van Inwagen (1990). It treats *part* as an undefined primitive notion and then defines all important notions in terms of just *part* and logical terms like *identity*. For example,

**x is a proper part of y**: x is a part of y and x ≠ y.

**x overlaps y**: there is a z such that z is a part of x and z is a part of y.

**y is a sum of the xs**: the xs are all parts of y; and every part of y overlaps at least one of the xs.

The definition of *proper part* entails that not all parts are proper parts. For example, everything is a part of itself, but nothing is a proper part of itself.

The definition of *overlap* effectively states that x overlaps y when they have a part in common. Two examples will help illustrate this concept. First, a covalent bond is formed when two atoms share electron pairs. In this case, the two atoms overlap, because both have the same electron pair as parts. Second, returning to the H_2_O example, the oxygen atom overlaps the H_2_O molecule, because both have the oxygen atom as parts. Recall that the oxygen atom is a part of itself.

The definition of *sum* can be illustrated as follows. *You* are a sum of your molecular parts because your molecules are all parts of you and every part of you (every simple, every molecule, every organ, etc.) overlaps at least one of your molecules.

We may ask what conditions must be obtained for there to be a sum. However, this is not the best way to capture the key question being addressed in this paper. To see why, consider that you are a sum of your simples *and* you are a sum of your molecules *and* you are a sum of your simples and your molecules (and your organs) all at once. It is unlikely that all these overlapping objects satisfy a finite non-trivial restriction on composition. At least, not one that can be easily described. So to formulate the central question, it helps to define:

**The xs compose y:** y is a sum of the xs and no two of the xs overlap.

We then have:

**The Composition Question**: What necessary and jointly sufficient conditions must any xs satisfy in order for it to be the case that there is an object composed of those xs?

We may now more carefully formulate the main answers:

**Nihilism**: For any non-overlapping xs, there is an object composed of the xs iff there is only one of the xs.

Nihilism allows that simples compose themselves. This is trivial composition. Nihilism rules out non-trivial composition: two (or more) non-overlapping xs never compose anything.

**Universalism**: For any non-overlapping xs, there is a y such that y is composed of the xs.

Consider a toy world with only two simples, *x*_1_ and *x*_2_. Nihilism states that only those two things exist. Universalism instead states that exactly three things exist, *x*_1_, *x*_2_, and the object they compose.

Let us now move away from the two extremes and define some restrictions on composition. Common sense suggests that the parts of a composite must be in some degree of contact. A simple hypothesis is then:

**Contact**: For any non-overlapping xs, there is an object composed of the xs iff the xs are in contact with one another.

While intuitive, it immediately faces *counterexamples*. The distance between the oxygen atom and one of the hydrogen atoms of an H_2_O molecule is about 95.84 picometers. Although small, the distance is non-zero, so the atoms are not strictly speaking in contact, so they do not compose a molecule after all. Indeed, it is unclear whether any two particles are literally in contact with each other, in which case, contact reduces to nihilism.

To resolve this issue, one might require only a certain degree of contact so that parts must be ‘close enough’. However, any exact distance measure will look arbitrary. But if the distance measure is vague, rather than exact, then we will be left with borderline or indeterminate cases of composition and therefore indeterminate cases of existence. This problem (the problem of *ontological vagueness*) and the problem of *counterexamples* are the two main problems faced by any restriction on composition.

One could replace contact with the requirement that parts of an object must simply be ‘fastened’, so that they move around together:

**Fastenation**: For any non-overlapping xs, there is an object composed of the xs iff the xs are fastened together.

Unfortunately, counterexamples are easy to come by (Van Inwagen’s amusing case involves two people who, while shaking hands, become paralyzed so that they are unable to pull their hands apart. Fastenation entails that a new object thereby comes into being, one composed of the two paralyzed handshakers. Or consider a calf that is ‘fastened’ to her mother). Indeed, universal gravitation suggests that all massive objects are fastened to some degree. Fastenation then collapses into universalism. We might then specify *a degree* of fastenation, but any such specification will seem arbitrary and *ad hoc* [Carmichael (2015) has defended the following restriction: for any non-overlapping xs, the xs compose y iff either (i) the xs are lump-like and the xs are bonded or (ii) the activities of the xs constitute an event that imposes sufficient unity on the xs. However, phrases like ‘sufficient unity’, ‘lump-like’, and ‘bonded’ are much too imprecise].

Finally, let us consider the two ideas that seem to us to hold the most promise. Merricks restricts composition to conscious beings, since he thinks causal irreducibility is essential to composition, and only conscious beings exhibit causal irreducibility. van Inwagen restricts composition to living beings, since he thinks self-maintenance is essential to composition, and only living beings seem to exhibit self-maintenance. Rather than formulating their views directly in terms of consciousness and life, we should formulate them in terms of the more fundamental notions:

**Causal irreducibility**: For any non-overlapping xs, there is an object composed of the xs iff either (i) the xs exhibit causal irreducibility or (ii) there is only one of the xs.

**Self-maintenance**: For any non-overlapping xs, there is an object composed of the xs iff either (i) the xs exhibit self-maintenance or (ii) there is only one of the xs.

van Inwagen requires condition (ii) because he requires simples: living things seem to have non-living parts. Merricks (2001, p116) argues that he does not require simples, since if matter were infinitely divisible, it would just follow that there are new levels of causal powers descending *ad infinitum*. We will see that IIT offers a novel way to think about simples. In what follows, we explain and defend the following proposal:

**Φ-restriction on composition**: For any non-overlapping xs, there is an object composed of the xs iff either (i) the xs are a complex i.e. they are a local maximum of Φ or (ii) the xs have non-zero *ϕ* relative to a complex.

## From causal irreducibility to integrated information

IIT postulates a mathematical measure of *amount of consciousness* in a system: the integrated information (Φ) of the system. A system with zero Φ is unconscious. A system that is a local maximum of Φ is conscious, and its level of consciousness corresponds to its level of Φ.

Integrated information (Φ) is really a measure of *amount of causal integration*. It is related to Merrick’s notion of causal irreducibility but does not require dualism [For an attempt to relate integrated information to causal irreducibility, see Hoel et al. (2016)]. Integrated information is associated with consciousness in part because of experimental evidence. In any realistic biological system that exhibits the signs of consciousness, exact computation of Φ is currently infeasible. However, the so-called ‘Φ-proxies’ have been shown to correlate with consciousness [There exist many ‘proxy’ measures of integrated information (Mediano et al. (2022)). Here it is useful to distinguish ‘IIT-inspired’ measures from ‘aspirational’ measures (Leung and Tsuchiya (2023)). The former are only weakly related to IIT. Examples are Massimini et al. (2005) and Casarotto et al. (2016), which at most provide evidence that a high level of consciousness depends on brain dynamics that (i) explore a large range of states, so that the current state can carry a large amount of information, and that (ii) exhibit integration of information between regions. Aspirational measures such as Afrasiabi et al. (2020) and Leung et al. (2020) are closer to the definition of Φ. Additionally, spatiotemporal patterns of integrated information have been derived from the fusiform gyrus and superior temporal gyrus and correlated with the quality (or contents) of conscious perception of faces and other objects (Haun et al. (2017)). Exact Φ calculations on simple networks can be performed at http://integratedinformationtheory.org/calculate.html. The software is described in Mayner et al. (2018)].

Instead of treating Φ as a measure of consciousness, we are exploring the idea that it is a restriction on mereological composition i.e. a criterion for when composites exist. However, we explore some possible connections to consciousness in the final section.

**Figure 1.**
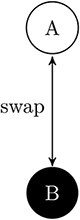
The simplest composite AB. A and B are simples. They can both take on one of two states (black=0) or (white=1). The laws governing their causal interactions make it so that they swap states at each time step. At the depicted time step, their state is [A=1, B=0]. So, the state at the previous, and at the next, time step is [A=0, B=1]. AB has four *possible* states 00, 01, 10, and 11. However, AB’s current state (and the causal laws) are only consistent with 01 being the previous and next state. The current state therefore contains *information* about its immediate past and future, which we can quantify: logarithm base 2 gives units of information such that inf(AB) = log_2_(4) - log_2_(1) = 2. So AB has two units of information about its past state and two units about its future state. This information is *integrated* because we would lose it by ‘noising’ the connections between A and B. A by itself gives zero information about A’s next (or previous) state. Same with B. So the integrated information that AB has about its past (and future) is Φ(AB) = inf(AB) - (inf(A) + inf(B)) = (2 - (0 + 0)) = 2. AB therefore exists.

The basic idea is this: if a system’s past/future states are specified (constrained) more by a composite than by its parts, this system can be considered as causally integrated and possessing of a certain amount of integrated information. To illustrate the idea, we will model the simplest non-zero Φ system, named AB, using an early mathematical formalism called IIT2.0. See *Figure 1*. IIT2.0 has been superseded by a more advanced formalism, IIT3.0. Still, IIT2.0 is much simpler and is useful for illustrating the following key concepts:

**Information**: A composite must contain *information* about its immediate past and future. For example, AB contains two units of information about its immediate past and two units of information about its immediate future.

**Integration**: A composite must contain information that is *integrated*. For example, cutting causal connections between A and B means AB no longer has information about its past or its future. This shows that AB’s information was integrated, since those connections made a difference. So, Φ(AB)=2.

**Figure 2.**
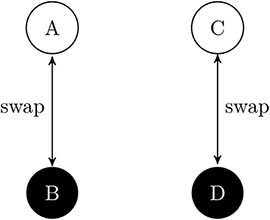
**A non-composite ABCD.** AB and CD are qualitatively identical. ABCD has 16 possible states: 0000, 0001, …, 0111, 1111. But the current state (and the causal laws) constrain the possible past and future states down to just one [0101]. The current state therefore contains information about its immediate past and future, which we can quantify: Inf(ABCD) = log_2_(16) - log_2_(1) = ( 4–0) = 4. From Figure 1 we know that inf(AB) = inf(CD) = 2. By identifying AB and CD as the appropriate parts of ABCD (see appendix for how to identify this), we have: Φ(ABCD) = inf(ABCD) - (inf(AB) + inf(CD)) = 0. ABCD therefore does not exist. Note that adding connections (e.g. between A and C or between B and D) may still result in Φ(ABCD) > 0, yet still ABCD may not form a composite. For example, we might add some connections and find some non-zero value for Φ(ABCD). But we still need to apply the exclusion postulate. If Φ(ABCD) < Φ(AB) or Φ(CD), then ABCD would still not exist as a composite: its Φ would be excluded due to the existence of composites AB and CD. If Φ(ABCD) > Φ(AB) and Φ(CD), then ABCD exists as a composite and depending on the connections, AB and CD may just be proper parts of ABCD (see Tononi (2004, figure 5)).

In *Figure 2*, a case of non-composition is illustrated. AB and qualitatively identical system CD are not causally interacting with each other, but they each exhibit self-interaction. We find that inf(ABCD) = inf(AB) + inf(CD), so the information in ABCD is reducible to the information in AB and in CD.

System ABCD can be used to illustrate a third crucial concept, that of *exclusion*:

**Exclusion**: A composite must be a local *maximum* of integrated information. This means it must have more Φ than any overlapping candidate object. A composite may therefore go out of existence if its Φ drops below the Φ of any overlapping candidate object. Prior to the connections between AB and CD being cut, ABCD may have existed. Its existence was then excluded by an overlapping system maximizing Φ.

Given some xs, a *candidate object* is any object recognized by universalism. For example, ABCD is a candidate composite object simply because we take the existence of A, B, C, and D for granted (perhaps they are simples). We can apply the IIT formalism to any xs to check whether they are more than just candidate proper parts.

Assume we add connections between A and C and between B and D, so that ABCD is an integrated system. The exclusion postulate entails that there are three different ways that ABCD’s existence could be excluded, despite having non-zero Φ. First, it might still be that ABCD’s existence is excluded by AB because Φ(AB) > Φ(ABCD). In that case, ABCD has been excluded by a (candidate) proper part. Second, it might be that ABCD is a proper part of some larger candidate ABCDEFGH such that Φ(ABCDEFGH) > Φ(ABCD). In that case, ABCD has been excluded by a (candidate) composite it is part of. Third, it might be that ABCD has proper parts in common with CDEF such that Φ(CDEF) > Φ(ABCD). In that case, ABCD has been excluded by a (candidate) composite it shares proper parts with.

IIT2.0 is helpful for introducing these key concepts. But it is not very helpful for mereology. In particular, it does not allow for the existence of any (non-trivial) composites that are themselves proper parts of composites. For example, take case two, where Φ(ABCDEFGH) > Φ(ABCD). Assuming that nothing excludes ABCDEFGH, we find that its only proper parts are the xs we assumed from the start, A, B, C, …, H. Similarly if ABCD exists then AB, AC, AD, etc. are all excluded. Obvious counterexamples arise. For example, the region of the brain that maximizes Φ would only have simples as proper parts: no neurons, no molecules, no atoms, etc. To remove these counterexamples, we need IIT3.0.

In IIT3.0, AB has one unit of Φ, not two (see appendix). More importantly, IIT3.0 introduces the concept of *ϕ* (small phi). Thus, for any composite that maximizes Φ, we can identify all (candidate) composites that it has as (candidate) proper parts. The candidates that really exist are those with non-zero *ϕ*. This concept is rigorously spelled out in the appendix, which considers the composite AB from above and finds that A and B both exist relative to AB, because they each have non-zero *ϕ*. Composites that maximize Φ are never proper parts of anything. For ease of exposition IIT refers to them as *complexes*, to distinguish them from composites that are proper parts. We therefore have our proposed restriction on composition:

**Φ-restriction on composition**: For any non-overlapping xs, there is an object composed of the xs iff either (i) the xs are a complex i.e. they are a local maximum of Φ or (ii) the xs have non-zero *ϕ* relative to a complex.

The procedure for calculating composites and their proper parts is computationally extraordinarily heavy and typically requires software even for small objects that consist of a few xs. For larger systems of interest, like people, trees, planets, etc., exact calculations are not currently feasible. Fortunately, for such systems, there are tools for making estimates, which we explain in the next section.

## From self-maintenance to feedback connectivity

Recall the *self-maintenance* restriction on composition: there is an object composed of the xs iff either (i) the xs exhibit self-maintenance or (ii) there is only one of the xs. van Inwagen held that self-maintenance is tied to life and postulated (ii) because living things should be ultimately composed of non-living things. Self-maintenance captures the idea of a system maintaining its existence over time. Due to special interactions among the system’s parts, the system is able to achieve an identity over time.

It is not clear what processes are necessary and sufficient for self-maintenance. However, *feedback connectivity* seems important [This may be illustrated by the Krebs cycle or by autopoiesis (in contrast with allopoiesis)]. This is a dynamic state of networks where causal connections between units of the network form a directed cycle. AB (from *Figure 1*) is an example feedback system. The directed cycle is formed because what A does to B influences what B does to A, and vice versa, over time.

Now consider a *feedforward* system, AB*, which is identical to AB except that B does not affect A. This means there is an arrow going from A to B, but no arrow going from B to A. Should AB* count as a composite? In a feedforward system, the causal input into the system is always determined entirely by external inputs and the causal outputs of the system never affect the rest of the system. There is a sense in which the system does not exist *for itself*: since its state at any time is entirely at the whim of the external, it can make no causal difference *to itself*. It therefore cannot maintain itself over time.

Feedback connectivity is essential to integrated information. The more feedback connectivity in a system, the more likely it is that the system maximizes Φ. This means we can use feedback connectivity estimates as a heuristic for identifying local maximums of Φ in very complex systems. We will exploit this heuristic when we consider possible counterexamples in section ‘The problem of counterexamples’. First, we need to acknowledge some limitations of IIT as it has so far been developed and how this relates to the challenge from ontological vagueness.

## The problem of vagueness

As discussed in the opening two sections, the self-maintenance restriction has been criticized on the basis of *ontological vagueness*, and many think that this criticism extends to any non-trivial restriction on composition. The general concern is that if the restriction is not well-defined, then we will be left with borderline cases of existence.

The problem for van Inwagen in particular is that because ‘life’ or ‘self-maintenance’ are vague terms, there will sometimes be no fact of the matter as to whether or not a given system is alive or self-maintains. These will be cases where there is no fact of the matter over whether composition occurs. But then there will be no fact of the matter over whether these things exist. This conflicts with the widely held view that things either exist or they do not.

Does the Φ-restriction solve the problem of ontological vagueness? It would appear to come closer to doing this than any other proposed restriction. For there are many interesting systems where IIT3.0 gives exact calculations and removes all vagueness. The problem, however, is that IIT3.0 is only mathematically defined for certain specific systems and not physical systems in general (Barrett and Mediano (2019)). In particular, IIT3.0 only applies to classical Markovian networks made up of interconnected units that interact with each other according to deterministic or probabilistic rules. Each unit can take on a number of states, and the state of the system is made up of the states of each of the units in the system.

In response, it has to be recognized that IIT is a work in progress that is constantly being updated to make it more general. It is based on a set of concepts (information, integration, exclusion, etc.) and a set of demonstrations of how those concepts can be mathematically formalized on certain sets of systems (IIT2.0 and IIT3.0). These can be seen as resources for generalizing IIT to a broader class of systems. Evidence that this can be done can be found in the recent extension of IIT3.0 to quantum mechanical networks, known as quantum integrated information theory (QIIT) [See Zanardi et al. (2018), Kleiner and Tull (2020), Chalmers and McQueen (2022). Some earlier attempts to give quantum physical definitions of Φ can be found in Tegmark (2015), Tegmark (2016), Kremnizer and Ranchin (2015)]. Thus, unlike other restrictions on composition, the Φ-restriction completely avoids vagueness for a particular class of systems, and there is an active research program aimed at extending IIT beyond such systems. This is the sense in which IIT makes significant progress in solving the vagueness problem.

Perhaps the biggest challenge to extending IIT, so that it gives mathematically exact verdicts for all physical systems, involves extending the formalism from discrete systems to continuous systems. This problem is especially pressing if fundamental physics ultimately describes reality entirely in terms of continuous fields (Barrett (2014)). This is not a problem that we aim to solve here. When considering challenging cases, we will simply assume that such cases can be thought of in terms of causally interacting discrete units. For example, in the case of the lake described in the next section, we can discretize it in terms of interacting portions of water. We may then consider, for example, how much feedback connectivity occurs among these candidate objects, relative to how much occurs at other scales, to determine whether the lake exists.

## The problem of counterexamples

We have seen that restrictions on composition tend to face counterexamples. Counterexamples to a proposed restriction are cases in which composition occurs according to the proposed restriction but *intuitively* should not occur (or composition does not occur according to the restriction but intuitively should occur). Mere intuition might seem like a blunt weapon to attack proposed restrictions with. But it must be kept in mind that a major motivation for such restrictions is to capture many ordinary intuitions about composition that both nihilism and universalism fail to capture. No restriction should be expected to perfectly capture everyone’s pre-theoretical intuitions about composition. But a good restriction should at least be able to explain composition well in a variety of challenging cases. Here, we argue that the Φ-restriction makes good sense of a variety of challenging cases. Calculation of Φ for most challenging cases is infeasible. However, we can make reasonable estimates by considering the mereological scale at which feedback connectivity is most prevalent.

### H_2_O molecules

In section ‘Formal statement of the composition question’ we began with the simple contact restriction and found that an H_2_O molecule would be a counterexample, since it intuitively exists yet its parts are not quite in contact. For van Inwagen, H_2_O molecules do not exist because they are not living. But do H_2_O molecules ever self-maintain themselves in the sense that the hydrogen and oxygen atoms exhibit feedback connectivity? This is a difficult empirical question. We suspect that an *isolated* H_2_O molecule has some small amount of Φ and so is a complex and that a non-isolated H_2_O molecule in a lake has some small amount of *ϕ*. In either case, H_2_O molecules exist.

### Lakes

Now consider trillions of such molecules, where the candidate composite of interest is a lake. Applying IIT to a lake means calculating the Φ of every possible set of particles in the vicinity of the lake and selecting the local maximums. Any organisms in the lake will count as local maximums, since their Φ will be significantly greater than the Φ of the lake as a whole. This does not exclude the lake however, which would not have the animals as parts anyway. There seem to be two interesting possibilities for the lake. One is that no set of H_2_O molecules in the lake generate more Φ than is generated by a single H_2_O molecule. In that case, what exist are each H_2_O molecule (they are complexes) and their parts, but no lake. The other is that the lake as a whole would have greater Φ than any of its candidate proper parts. In that case, the lake (along with the organisms swimming in it) all exist. For now, it is too difficult to estimate which of these two possibilities are obtained. Nonetheless, this illustrates IIT’s novel take on composites: whether a given candidate composite exists (as a complex or as a proper part of one) depends on the overall *state* of the network it is causally embedded in. And since the states of networks can fluctuate, so too can the mereological status of any candidate object in that network.

### Classrooms

Intuitively, the students in a classroom all exist, but there is no object composed of the students. Of course, in a well-taught classroom, the students will frequently be giving each other feedback. The students collectively will then be a possible candidate system with non-zero Φ. However, the feedback connectivity among students pales in comparison to the feedback connectivity inside a student’s brain (Shehata et al. (2021)). The student brains (and therefore, the students) exclude the classroom composite in favor of the students. See also Engel and Malone (2018).

### Forests

On the face of it, trees in a forest do not interact much. It seems that most feedback connectivity in a forest happens inside individual trees and is responsible for their growth. Trees even have brain-like properties, such as action potentials in their root tips and the use of gap junction membrane potentials (Canales et al. (2018)). In that case, the trees are analogous to the students, and the forest is analogous to the classroom. However, recent studies have found significant feedback connectivity between trees in a forest. Trees of the same species are communal but also form alliances with trees of other species. This is possible because trees are connected to each other through underground fungal networks (Simard et al. (2012)). These networks allow trees to share water and nutrients and to communicate information about insect attacks, disease, drought, and so on. Of course, students communicate too but are not excluded by any network supporting that communication. So here, it is entirely unclear what maximizes Φ in a forest. Is it the forest, the trees, or the tree’s cellular parts? This is an open question.

### Starling murmurations

Murmurations are huge groups of starling birds that twist, turn, swoop, and swirl across the sky in beautiful shape-shifting clouds. Scientists still are not sure how each starling knows which way to turn without bumping into the others. However, it was recently found that each starling seeks to match the direction and speed of the nearest seven or so neighbors, rather than responding to the movements of all of the nearby birds around them (Ballerini et al. (2008)). This yields significant feedback connectivity. Arguably, however, the neural network of any bird brain would still yield greater Φ than the murmuration of birds. For while a bird may exhibit feedback connectivity with seven or so other birds, a bird brain neuron will exhibit feedback connectivity with 1000–10 000 neurons if it is a typical mammalian brain. Thus, it seems the starlings exist and their existence excludes that of the murmuration. A related issue applies to schools of fish—for a recent IIT-related analysis see Niizato et al. (2020).

### The internet

Could human beings ever integrate themselves into a system that has greater integration than any human brain? The human brain is the most integrated system we know of, but supercomputers may soon be able to simulate the complexity of the brain. The internet itself is an extremely interconnected system with great potential, thus it is theoretically possible. However, the complexity of the internet is constrained by the needs of its users. If the internet’s interconnectivity gets too complex, it may be difficult to control and use effectively. Thus, internet users may not want an internet whose Φ is greater than the Φ of a brain. Still, if it were to happen, would our existence be excluded? Probably not, since we expect human brains to have non-zero *ϕ* relative to the internet complex.

### Tables and chairs

As a final challenge, consider inanimate objects, like tables and chairs. On the one hand, there is a strong intuition that these objects exist and so should be captured by any proposed restriction on composition. On the other hand, it seems unlikely that a table is a maximum of Φ. No matter what scale we consider—portions of wood, molecules, and atoms—there seems to be little feedback connectivity. It might turn out that feedback connectivity is maximized by particular molecules in the table. In that case, the molecules exist, but they do not compose a further object, the table. Would this result be enough to refute our proposed restriction? We think not. First, the strong intuition is based on day-to-day familiarity, whose metaphysical relevance is questionable. Second, the Φ-restriction is itself based on a metaphysical intuition, whose starting point is a metaphysical principle with a long history, the so-called Eleatic Principle: to be is to have causal powers (Colyvan (1998)). IIT adds that that composites must have causal powers over themselves, as measured by their integrated information. Insofar as inanimate objects do not have such powers, the intuition based in the Eleatic Principle may be seen to override the everyday intuition. Finally, inanimate objects of course pose a problem for van Inwagen’s life restriction, but van Inwagen resolved it as follows: the challenge is really just a matter of making sense of the *correctness* of statements such as ‘there is a table in the room’, which are literally false if there are no such composites. Such statements are correct because there is a true statement in the vicinity: there are some xs in the room and they are ‘arranged chairwise’ van Inwagen (1990, 108-111). Thus, it is open to our account to adopt van Inwagen’s paraphrase strategy.

## Conclusion

As Lewis (1986, 213) put the central challenge to composition restrictions: ‘no restriction on composition can be vague, but unless it is vague, it cannot fit the intuitive desiderata. So no restriction on composition can serve the intuitions that motivate it’. Here we have argued that the Φ-restriction goes a long way in solving the vagueness problem, while offering reasonable explanations of a variety of challenging cases. IIT should therefore be considered the front-runner among answers to the composition question that propose restrictions on composition.

We conclude by considering possible implications of our proposal for consciousness and existence. We have so far interpreted Φ (and *ϕ*) as a criterion for the existence of composite objects: for any non-overlapping xs, there is an object composed of the xs iff either (i) the xs are a complex i.e. they are a local maximum of Φ or (ii) the xs have non-zero *ϕ* relative to a complex. Our paper is neutral on whether Φ is also a criterion for consciousness. However, under its usual interpretation, IIT does indeed make Φ a criterion for consciousness: when the xs are a local maximum of Φ, the object they compose is conscious. Indeed IIT goes further and postulates that consciousness comes in *degrees*: the amount of consciousness in a conscious system corresponds to its amount of Φ. If we take seriously the idea that Φ might also be a criterion for consciousness then there are several interpretations of our framework. Following Lee (2022), we can say that if something comes in degrees, then it is *degreed*; if not, then it is *dichotomous*. Our framework is then consistent with the following interpretations:

1. Existence and consciousness are dichotomous.
2. Existence is dichotomous and consciousness is degreed.
3. Existence is degreed and consciousness is dichotomous.
4. Existence and consciousness are degreed.

Interpretations (1) and (2) follow our approach by treating Φ (and *ϕ*) as the criterion for when composition occurs, without implying that composites with more Φ exist to a greater degree. The idea that existence comes in degrees is widely rejected in philosophy. For what could it possibly mean for one composite to exist to a greater degree than another? Indeed, given that human brains appear to have the greatest degree of Φ of any composites we know of, interpretations (3) and (4) would entail that human brains enjoy the greatest degree of existence. It is not clear what this means. Still, those who argue that we have independent reason to believe that existence comes in degrees (e.g. McDaniel (2013)) may find our approach useful, since it is possible to treat Φ as a measure of the degree to which a composite exists.

Interpretations (1) and (3) treat consciousness as dichotomous. Although this is not the standard IIT approach it may be favored by those who are skeptical that consciousness comes in degrees (e.g. Pautz (2019)). It is possible to drop the hypothesis that consciousness comes in degrees from IIT and just treat Φ as the criterion for when something is conscious (McQueen (2019b, sec. 2.2)). Note that panpsychism is not an implication of any of these approaches, since in IIT, the xs compose a conscious being only when they are a local maximum of Φ. Merely having non-zero *ϕ* relative to a complex is insufficient for having consciousness, but it is sufficient for existence. In IIT, the *ϕ* of an object O helps to characterize the quality of the consciousness of the conscious complex that has O as a proper part. It does not also measure the quantity of consciousness in O, since O’s consciousness is excluded by the complex.

## Figures and Tables

**Figure A1.**
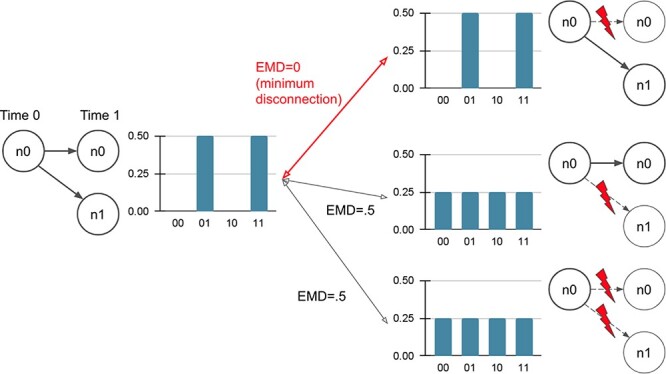
n0’s future influence (effect). Among n0’s potential candidate purviews, {n0, n1} has 0 influence as there is a minimal disconnection from n0 to n0 (top, right) that does not affect anything on the probability distribution of the system’s state in the future.

**Figure A2.**
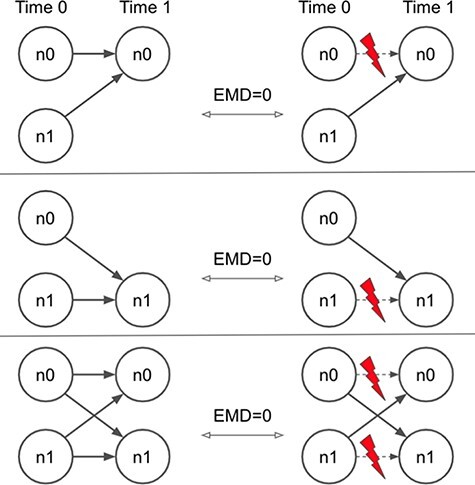
For candidate part {n0, n1}, each potential purview has a minimal disconnection that does not affect the future state of the system. Thus, {n0, n1} as a candidate part does not have any effect into the future of this system (zero effect).

**Figure A3.**
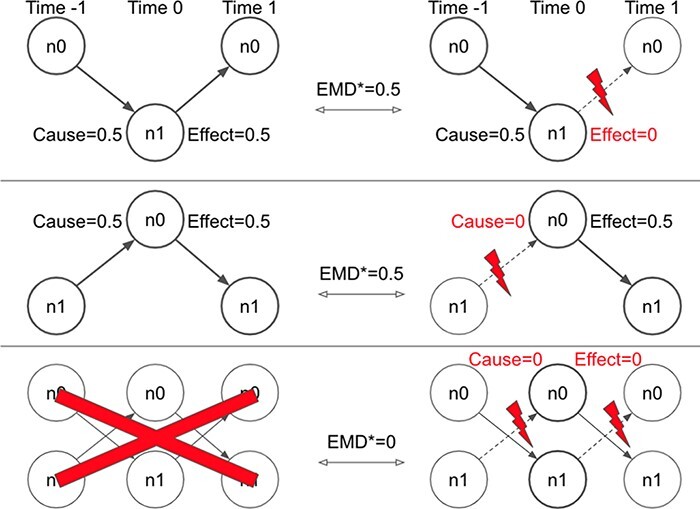
To assess the degree of the existence of a system, we compare the set of probability distributions for the original system (left) and the minimally unidirectionally disconnected system (right). Upon the disconnection, n1 has 0 effect and n0 has 0 cause, thus they do not exist. {n0, n1} also has minimal disconnection into its future and past purview, making its effect and cause to be 0. Thus, {n0, n1} also does not exist upon the disconnection. In sum, with a system-level minimal disconnection, nothing exists. The (generalized) earth mover’s distance (EMD*) between the two distributions (left and right) arrives at a degree of integrated information of 1.

## Appendix A How to calculate composites with IIT3.0

In this appendix, we explain how the latest version of IIT (3.0) analyzes the two-element system that we featured in the main text using the former IIT formalism (2.0) [Chalmers and McQueen (2022) in their appendix also provide IIT3.0 calculations for this same system. However, their emphasis is on calculating the ‘Q-shape’ of the system, whereas our emphasis is on identifying parts and wholes ].

There are several key differences between 2.0 and 3.0. We do not cover all the differences and point out only those that are critical to understanding how 3.0 differs from 2.0. The order of the differences that we explain does not imply the importance of the differences.

First, 3.0 considers the influence of (candidate) parts to the whole system. Second, 3.0 analyzes the constraints that the current state of a system has over its past state (e.g. what could have caused the current state). Likewise, it analyzes the constraints that the current state has over its future state (e.g. what could happen in the future based on the current state). Third, 3.0 searches for all possible scopes of influence (called ‘purview’) that (candidate) parts can have and takes the maximum (called core causes or core effects). We will explain each of these as we explain how 3.0 analyzes the two-element system.

We assume that the considered world consists of the two-element system and nothing else. This greatly simplifies the computation [We attach the pyphi code here (made by Yota Kawashima): https://github.com/yotaKawashima/PyphiSample. Consistent with this code, we now refer to elements A and B (from section ‘From causal irreducibility to integrated information’) as elements n0 and n1]. The analysis starts from characterizing how the system transits from one state to another, for each possible state of the system, using the transition probability matrix (TPM). The TPM of this system is shown in *Table A1*.

**Table A1.** The TPM of the two-element (n0 and n1) system. Each element in the system copies its state to the other element over one time step. The left column describes the state of the system in each row at time *t* = 0. The middle and right columns describe the probability of each element’s state becoming 1 at the next time step (*t* = 1). This TPM describes all relevant causal properties of the system, both at the candidate parts level (n0 and n1) and the candidate composite level (n0 and n1).

[n0, n1]	Prob($n0=1$)	Prob($n1=1$)
[0, 0]	0	0
[1, 0]	0	1
[0, 1]	1	0
[1, 1]	1	1

### The existence of a candidate part, n0

First, we will consider the effect that a candidate part n0 has on the whole system. To start, list n0’s scope of possible future influences (future purview) as {{n0,n1}, n0, n1}. Let us take {n0, n1} as an example. To quantify the magnitude of irreducible influence from n0 to {n0, n1}, we consider what would happen if we ‘minimally’ disconnect n0 from {n0, n1}. (Note that ‘disconnection’ in IIT means replacing the connection with noise). There are three candidate ways to disconnect n0 from {n0, n1}, that is, disconnect n0 from n0, n1, or both n0 and n1. Among those, the *minimal* disconnection turns out to be from n0 to n0. Why? As there is no connection from n0 at *t* = 0 to n0 at *t* = 1 to begin with, there is nothing to ‘replace’ with noise, thus no effects. In other words, the probability distribution over the future purview does not change when we minimally disconnect n0 (at *t* = 0) from n0 (at *t* = 1). IIT considers this case as 0 irreducible influence (effect) from n0 to the candidate future purview {n0, n1}. Similarly, there is 0 cause from n0 to the candidate past purview n0.

Now, what happens if we consider candidate future purview n1? There is only one way to disconnect n0 from n1. And after the disconnection, there is no way to predict the state of n1 in the future. This gives a flat probability distribution over n1’s possible future states. As a consequence, IIT quantifies the influence of the minimal disconnection from n0 to the future candidate purview n1 to be *the difference in probability distribution* between the original distribution and the after-disconnection distribution. The so-called ‘earth mover’s distance’ quantifies this difference as 0.5. (The procedure to arrive at 0.5 is effectively the same as in Figure A1 when we considered the effect of the n0 to n1 cut).

Among all these three purviews, n0’s ‘core’ effect is n1. This intuitively makes sense as n0 is indeed affecting n1 only. Now, n0’s cause can be similarly analyzed to arrive at its ‘core’ cause, which is n1 and its magnitude is 0.5. IIT then proposes that n0’s amount of integrated information is to be the minimum of the cause and effect, that is, 0.5 in this case. We can perform the same analysis to quantify n1’s integrated information, which is 0.5, with its core cause and effect being n0. Both parts therefore exist.

### {n0, n1} does not exist as a part according to IIT3.0

Next, we perform the same analysis as above on the candidate composite part, {n0, n1} (*Figure A2*). Here we are effectively considering whether or not {n0, n1} exists as a part *of itself*. Perhaps, surprisingly to some, we find out that {n0, n1} does not exist as a part of the whole two-element system. (Although, as we explain below, {n0, n1} does exist *as a composite*.)

We first consider {n0, n1}’s possible future purviews, {n0, n1}, n0, n1. In each case, it turns out that there are minimal disconnections that do not affect the future probability distribution over the system’s state space. Thus, {n0, n1}’s future purview is undefined with 0 effect.

The same argument holds with {n0, n1}’s core cause (undefined purview with 0 cause). Thus, the minimum of cause and effect is 0, meaning {n0, n1} does not exist as a part.

### The existence of the two-element system as a composed entity

Finally, IIT considers how the two-element system of n0 and n1 exists as a composite by assessing the irreducibility of the system itself (*Figure A3*). To consider this, IIT introduces system-level unidirectional disconnections. In our two-element system, this disconnection is either from n0 to n1 or from n1 to n0. Both give the same result. Let us consider the disconnection from n0 to n1.

This disconnected system has only unidirectional influence from n1 to n0. We can repeat the same procedure above (i.e. specifying the TPM of the system with only connection from n1 to n0, which copies n1’s state into n0, going through purviews for each candidate system, etc.). In this case, it is easy to see that all three candidate parts vanish.

For n0, as it does not have influence to the future, its core effect is 0. Thus, the minimum of the cause and effect is 0. Thus, n0 does not exist for this disconnected system. For n1, as its cause does not exist, n1 does not exist. For {n0, n1}, the disconnection depicted in Figure A2 arrives at 0 effect and 0 cause. As a result, this disconnected system has no existence whatsoever at any level of the analysis.

The last step to quantify the degree of integrated information of the whole two-element system is to measure the distance of cause–effect probability distribution of the original system and the minimally disconnected system. IIT3.0 proposes that we can estimate it as the weighted earth movers’ distance (or generalized EMD, EMD*). For example, distribution into the future purview of the part n0 in the original system is moved into null distribution (0.25 for all states), weighted by the degree of integrated information of n0 (which is 0.5 in this case). For n0’s past, this corresponds to 0.5 × (0.25 + 0.25) = 0.25. Similarly, for n0’s future, we have 0.25. We do the same with n1, and that is it for this system. In total, we quantify the degree of integrated information of this composite to be Φ=1, establishing its existence.
